# Unmasking Cryoglobulinemia: A Cold-Blooded Complication of Hepatitis C

**DOI:** 10.1155/crhe/8382433

**Published:** 2025-07-24

**Authors:** Alexandra M. Arges, Ari Levine

**Affiliations:** Department of Medicine, Emory University Hospital, Atlanta, Georgia, USA

## Abstract

A 43-year-old man presents to the hospital with two weeks of persistent fevers, accompanied by myalgias, hematochezia, and abdominal pain. Acute infectious causes were ruled out, and elevated inflammatory markers suggested inflammatory diarrhea or autoimmune conditions. Esophagogastroduodenoscopy (EGD) and colonoscopy were negative. Further testing showed positive antinuclear antibodies (ANAs), ribonucleoprotein (RNP), rheumatoid factor (RF), and hepatitis C virus (HCV) RNA, suggesting an HCV-associated autoimmune process. Hematuria and neuropathic pain raised suspicion for mixed cryoglobulinemia secondary to HCV, supported by low complement levels. Treatment started with prednisone. Cryoglobulins came back positive, confirming mixed cryoglobulinemia secondary to HCV. The patient was referred to the hepatology clinic for antiviral treatment, where he completed treatment, with symptoms resolving, except for his neuropathy.

## 1. Introduction

Hepatitis C affects ∼71 million people globally, with approximately 1.5 million new infections annually [1, 2]. Approximately 80% of acute hepatitis C cases are asymptomatic, while the rest show fever, fatigue, and gastrointestinal symptoms. Up to two-thirds of the cases have extrahepatic manifestations, including mixed cryoglobulinemic vasculitis, B-cell lymphoma, autoimmune cytopenia, hypothyroidism, cardiovascular disease, and type 2 diabetes [2]. When approaching fever of unknown origin, we often prioritize ruling out infectious etiologies, including the viral hepatitides [3]. However, due to acute hepatitis C rarely being symptomatic, a clinician should inquire how chronic hepatitis C could lead to persistent fevers and consider an autoimmune etiology, such as mixed cryoglobulinemic vasculitis.

## 2. Case Presentation

A 43-year-old male presented to the hospital with two weeks of fevers, myalgias, diarrhea, and bloody stools. He denied any rashes, chest pain, or shortness of breath. Social history was notable for no intravenous drug use, heavy alcohol use, or tobacco use. There was no known personal or family history of autoimmune disease or known inflammatory bowel disease. On examination, he was hypertensive, tachycardic, and febrile, with periumbilical tenderness to palpation and submental cervical lymphadenopathy. As shown in Table 1, the patient's labs showed elevated liver enzymes (AST/ALT), elevated white blood cell count (WBC), elevated C-reactive protein (CRP) and erythrocyte sedimentation rate (ESR), and positive hepatitis C antibodies. Initial imaging was unremarkable.

On day two, he continued to experience fevers, yellow stools, and abdominal pain. The infectious workup was negative, and attention shifted to the possibility of inflammatory diarrhea. Tests for autoimmune conditions were ordered due to elevated ESR, CRP, and ferritin, as shown in Table 1.

**Figure 1:**
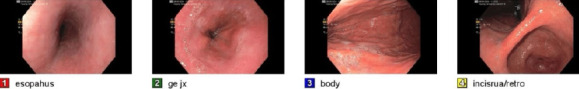
EGD, part 1. The examined esophagus was normal. Esophagogastric landmarks were identified: the gastroesophageal junction was found at 34 cm from the incisors. A few dispersed small erosions with no stigmata of recent bleeding were found in the *gastric body* and in the *gastric antrum.* Multiple biopsies were obtained on the greater curvature of the gastric body, on the lesser curvature of the gastric body, at the incisura, on the greater curvature of the gastric antrum, and on the lesser curvature of the gastric antrum with cold forceps for histology. The examined duodenum was normal. Multiple biopsies were obtained in the duodenal bulb and in the second portion of the duodenum with cold forceps for histology.

**Figure 2:**
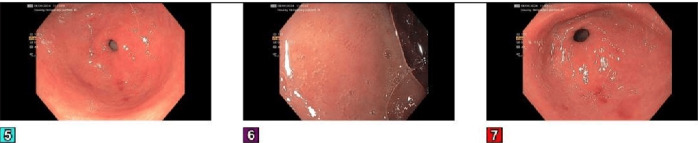
EGD, part 2. Gastric antrum erosions seen in pictures 5 and 7.

**Figure 3:**
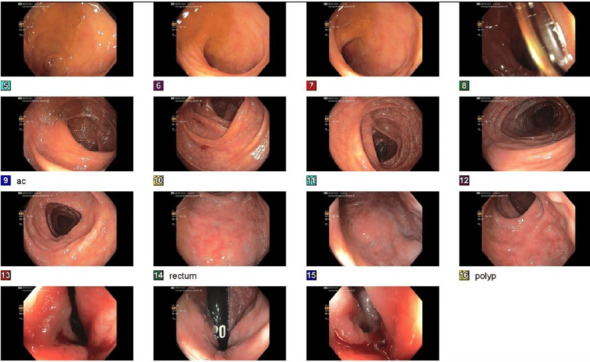
Colonoscopy. The perianal and digital rectal examinations were normal. The terminal ileum contained a single small diverticulum. The remainder of the exam in the terminal ileum was normal. A patchy area of mildly erythematous mucosa was found in the rectum, probably prep induced. A 2-mm polyp was found in the rectum. The polyp was sessile. The polyp was removed with a cold biopsy forceps. Resection and retrieval were complete. The exam was, otherwise, normal throughout the examined colon.

By day three, symptoms persisted, and additional labs, as shown in Table 1, revealed positive ANA, RNP, and RF, as well as elevated hepatitis C RNA PCR, raising concerns for a hepatitis C-linked autoimmune process. Urinalysis, as shown in Table 1, also revealed hematuria, suggesting possible glomerular involvement. Esophagogastroduodenoscopy (EGD) and colonoscopy, as shown in Figures1, 2, and 3, revealed erosive gastropathy as well as erythematous rectal mucosa but lacked overt abnormalities to explain his symptoms.

**Figure 4:**
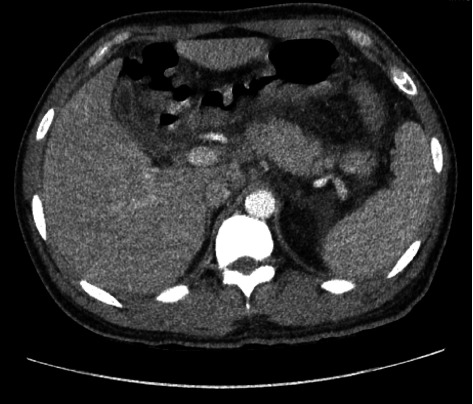
CT Abdomen/pelvis. Diffuse mesenteric edema and fat stranding seen throughout the visualized upper abdomen which could be reactive in the setting of active hepatitis infection.

On day five, the patient developed neuropathic pain in his feet, and mixed cryoglobulinemia secondary to hepatitis C was suspected. On day six, with our patient having persistent symptoms, low C3 and C4 levels, and a pending cryoglobulin test, mixed cryoglobulinemia was highest on our differential. CT imaging, as shown in Figure 4, revealed trace pleural effusion and mesenteric edema, which have been observed in the setting of cryoglobulinemia. The patient was started on prednisone 60 mg daily for suspected cryoglobulinemia and gabapentin for neuropathy, and plans were made for outpatient follow-up at a hepatitis C clinic.

On day seven, cryoglobulins were confirmed positive, as shown in Table 1, clinching the diagnosis of mixed cryoglobulinemia secondary to hepatitis C. The patient's neuropathy improved with steroids and gabapentin, and he was discharged to home, with close follow-up in hepatology clinic. Great efforts were made to try and initiate an antiviral in the inpatient setting, but these medications were not on formulary, and infectious disease consultants recommended initiation in the clinic, where his laboratory values could be monitored.

The patient followed up in clinic upon discharge and completed 8 weeks of Mavyret (glecaprevir/pibrentasvir), antiviral therapy for hepatitis C. His hepatitis C viral load was undetectable after 8 weeks of antiviral treatment. His symptoms of fevers, headache, malaise, arthralgias, and cough resolved. However, he continued to experience neuropathy in his feet, which improved with gabapentin.

## 3. Discussion

We present a case of HCV-associated mixed cryoglobulinemia that manifested as fever of unknown origin with predominant gastrointestinal symptoms and lacking a petechial rash. This case serves as a reminder to consider extrahepatic manifestations of HCV even in the absence of characteristic symptoms.

Mixed cryoglobulinemia is an extrahepatic complication of HCV resulting in proteins that precipitate from a patient's serum or plasma at temperatures lower than 37°C [2, 4, 5]. Reported prevalence in patients with HCV is between 40% and 65% [4]. The most common presenting symptom is a purpuric rash [2, 4, 5]. GI involvement, arthralgias, peripheral neuropathy, and glomerulonephritis can also be present [2, 4, 5].

Mixed cryoglobulinemia (types II and III) presents with polyclonal IgG and IgM with elevated rheumatoid factor (RF) and often with low C4 and normal C3 [4, 5]. Meltzer's triad of purpura, fatigue, and arthralgias appears in 80% of the cases, with purpura being the most common symptom [4]. Peripheral neuropathy is also seen [2, 4, 5]. The most common neurological consequence of HCV is peripheral neuropathy, with approximately 10% of patients with HCV having clinically symptomatic sensory or motor peripheral neuropathy, and approximately 90% of the patients with HCV-associated mixed cryoglobulinemia having neuropathy [6]. Renal involvement in mixed cryoglobulinemia shows a membranoproliferative glomerulonephritis detected through renal failure or nephritic syndrome [2, 4]. Management varies with severity, from antivirals in mild cases to IV steroids and plasma exchange for severe cases [2, 4]. Access remains a challenge for HCV treatment, with treatments lasting 8–12 weeks [1]. Hepatitis C is not typically associated with acute febrile illness, but mixed cryoglobulinemia should be considered if neuropathic pain, high inflammatory markers, positive RF, and cryoglobulins are present.

## 4. Conclusions

Hepatitis C is not typically associated with acute febrile illness, but mixed cryoglobulinemia should be considered if neuropathic pain, high inflammatory markers, positive RF, and cryoglobulins are present. Treatment for mixed cryoglobulinemia involves treating the underlying cause (in this case, HCV, with antivirals) and calming the immune response with steroids, rituximab, or even plasmapheresis. Peripheral cryoglobulin-related neuropathy in HCV remains difficult to treat, with some trials showing success with rituximab or plasmapheresis [5, 6].

## Figures and Tables

**Table 1:** Laboratory values.

Parameters	Patient values	Reference range
Sodium (mmol/L)	133	136–145
Potassium (mmol/L)	4.4	3.5–5.1
Chloride (mmol/L)	105	98–107
Carbon dioxide (mmol/L)	18	23–29
Calcium (mg/dL)	8.6	8.6–10.3
Blood urea nitrogen (mg/dL)	56	7–25
Creatinine (mg/dL)	1.5	0.7–1.3
Glucose (mg/dL)	125	70–105
Alkaline phosphatase (unit/L)	73	34–104
Alanine aminotransferase (unit/L)	81	7–52
Aspartate aminotransferase (unit/L)	81	13–39
White blood cell (10E3/mcl)	20	4.2–9.1
Hemoglobin (gm/dL)	12.3	12.9–16.1
Hematocrit (%)	37.1	37.7–46.5
Platelet (10E3/mcl)	172	150–400
C-reactive protein (mg/L)	74	< 10
ESR sedimentation rate (mm/hr)	40	1–15
Ferritin (ng/mL)	460	24–336
ANA (antinuclear antibody) titer	1:160	< 1:80
Rheumatoid factor (RF) IU/mL	5.1	0–3.5
Ribonucleoprotein IgG AI	4	0–0.9
Hepatitis C antibody	Positive	Negative
Hepatitis C viral RNA quant by PCR (IU/mL)	278,000	0–14
C3 complement level (mg/dL)	57	81–157
C4 complement level (mg/dL)	3	13–39
Protein urine qualitative mg/dL	50	Negative
Blood urine qualitative	Moderate	Negative
RBC/HPF urine	24	Negative
Cryoglobulin	Positive	Negative

*Note:* Summary of pertinent laboratory investigations from the case report.

## Data Availability

The data that support the findings of this study are available from the corresponding author upon reasonable request.
